# Chemical Modification of Nanocrystalline Cellulose for Manufacturing of Osteoconductive Composite Materials

**DOI:** 10.3390/polym16131936

**Published:** 2024-07-06

**Authors:** Olga Solomakha, Mariia Stepanova, Anatoliy Dobrodumov, Iosif Gofman, Yulia Nashchekina, Alexey Nashchekin, Evgenia Korzhikova-Vlakh

**Affiliations:** 1 Institute of Macromolecular Compounds, Russian Academy of Sciences, St. Petersburg 199004, Russia; solomanya@bk.ru (O.S.); anatoliy.dob@gmail.com (A.D.); gofman@imc.macro.ru (I.G.); 2 Institute of Cytology, Russian Academy of Sciences, St. Petersburg 194064, Russia; ulychka@mail.ru; 3 Ioffe Institute, St. Petersburg 194021, Russia; nashchekin@mail.ioffe.ru

**Keywords:** nanocrystalline cellulose, heparin modification, poly(glutamic acid), cellulose functionalization, poly(*ε*-caprolactone), polymer composites, osteoconductive materials

## Abstract

Cellulose is one of the main renewable polymers whose properties are very attractive in many fields, including biomedical applications. The modification of nanocrystalline cellulose (NCC) opens up the possibility of creating nanomaterials with properties of interest as well as combining them with other biomedical polymers. In this work, we proposed the covalent modification of NCC with amphiphilic polyanions such as modified heparin (Hep) and poly(*αL*-glutamic acid) (PGlu). The modification of NCC should overcome two drawbacks in the production of composite materials based on poly(*ε*-caprolactone) (PCL), namely, (1) to improve the distribution of modified NCC in the PCL matrix, and (2) to provide the composite material with osteoconductive properties. The obtained specimens of modified NCC were characterized by Fourier-transform infrared spectroscopy and solid-state ^13^C nuclear magnetic resonance spectroscopy, dynamic and electrophoretic light scattering, as well as thermogravimetric analysis. The morphology of PCL-based composites containing neat or modified NCC as filler was studied by optical and scanning electron microscopy. The mechanical properties of the obtained composites were examined in tensile tests. The homogeneity of filler distribution as well as the mechanical properties of the composites depended on the method of NCC modification and the amount of attached polyanion. *In vitro* biological evaluation showed improved adhesion of human fetal mesenchymal stem cells (FetMSCs) and human osteoblast-like cells (MG-63 osteosarcoma cell line) to PCL-based composites filled with NCC bearing Hep or PGlu derivatives compared to pure PCL. Furthermore, these composites demonstrated the osteoconductive properties in the experiment on the osteogenic differentiation of FetMSCs.

## 1. Introduction

Cellulose is one of the main natural polysaccharides that is derived from renewable sources in the biosphere, such as plants, algae and some bacteria [1,2,3]. Besides its renewability and abundance, cellulose combines chemical inertness with the potential for modification and functionalization [4,5]. Currently, cellulose has a wide range of industrial applications [6,7,8]. Furthermore, micro- and nanoparticles of cellulose, due to their physicochemical properties, are considered as an independent useful class of materials [9], as well as a filler for the production of polymer composites [10]. Cellulose micro- and nanomaterials exhibit excellent stiffness, high strength, low coefficient of thermal expansion, low density, dimensional stability, high hydrophilicity and biocompatibility. 

The unique properties of micro- and nanocrystalline cellulose (MCC and NCC) make them very attractive for the preparation of biomaterials [11,12,13]. For example, NCC hydrogels are studied as implantable thermoresponsive materials [14,15], materials with pH-sensitive drug release [16,17] or bioinks for 3D-printing of scaffolds for tissue engineering [18]. In addition, the combination of NCC with other natural polymers such as gelatin [19], chitosan [20], alginate [21], hyaluronic acid [22], etc., *via* chemical or physical crosslinking allows the development of drug delivery systems and scaffolds for soft tissue engineering. Other applications of NCC/MCC for the production of scaffolds involve cross-linking NCC with poly(lactic acid)-based particles [23] and using them as fillers for biodegradable materials based on aliphatic polyesters [24,25].

In order to obtain chemically cross-linked materials based on NCC/MCC or to provide a particular functionality to the filler, NCC/MCCs are modified using a number of various techniques [26]. Covalent modification of NCC/MCC involves reactions of cellulose functional groups, namely hydroxyls. Depending on the modifying agents, the chemical reactions of cellulose hydroxyls may result in the formation of ester, urethane or silyl ether bonds [27,28]. To introduce into cellulose the unsaturated groups suitable for (1) radical copolymerization, (2) thiol-ene click reactions or (3) reactions with maleimide-containing components, typically, NCC/MCC is first modified with (meth)acrylic acid [23,26,29,30]. 

Partial hydrophobization of NCC/MCC is a key route to improve the compatibility of the hydrophilic filler with aliphatic polyesters [26,31,32]. In this case, NCC is modified by esterification, acylation or silanization with corresponding compounds containing long aliphatic or aromatic moieties. In particular, modification of NCC/MCC with butyl, hexyl, dodecyl or benzoyl moieties promotes homogeneous distribution of the filler in a matrix based on poly(lactic acid) (PLA) or poly(*ε*-caprolactone) (PCL) and, as a consequence, improves the mechanical properties of the composite materials [26]. Moreover, the introduction of NCC/MCC into polyester materials improves their hydrophilization and, consequently, cell adhesion and proliferation. In addition, the introduction of such filler affects the biodegradability of such materials. For example, PCL filled with NCC/MCC showed enhanced matrix degradation [26,33], which is explained by the following processes. At the initial step of polymer degradation, the hydrophilic filler can be released from the hydrophobic matrix due to surface erosion. The latter is accompanied by an increase in the specific surface area, owing to the expansion of the porosity of the scaffold walls. As a result, diffusion of water and enzymes inside the scaffold is facilitated. This in turn promotes non-enzymatic hydrolysis as well as adsorption of enzymes and further enzyme-mediated degradation.

Besides small hydrophobic radicals, grafting hydrophobic or amphiphilic oligomers or polymers from/to NCC/MCC has been reported to improve the compatibility of NCC/MCC with aliphatic polyesters. Among such modifiers, PLA [34,35], PCL [36,37] and poly(methyl methacrylate) (PMMA) [38] grafted from NCC/MCC, and amphiphilic oligomers of glutamic acid [39] grafted to NCC, have been used to modify cellulose and prepare composites with aliphatic polyesters. However, while the modification of PLA, PCL and PMMA only affects the improvement of interfacial biocompatibility of the filler with the hydrophobic matrix polymer, modification with amphiphilic polypeptides can specify biological functionality and provide composites with additional properties. For example, the use of NCC modified with amphiphilic oligomers of glutamic acid as a filler for PCL [40] improves biocompatibility of the materials [41] and promotes bone regeneration *in vivo* through additional osteoconductive properties [27]. The effect of glutamic acid peptides on calcium phosphate nucleation and osteogenic differentiation of marrow stromal cells was recently shown by Karaman et al. [42].

In this study, we proposed two novel strategies to modify NCC with poly(glutamic acid) (PGlu) derivatives. Specifically, PGlu containing units with *γ*-benzyl protective groups (P[Glu-*co*-Glu(OBzl)]) and additionally modified with lysine (Lys) (P[Glu-*co*-Glu(Lys)-*co*-Glu(OBzl)] were used to functionalize NCC. Furthermore, a route for modifying NCC with a functionalized heparin (Hep) derivative was also proposed. As (poly)peptides of glutamic acid, Hep is a biocompatible and biodegradable polyanion. It possesses broad biological activities, such as anticoagulant, anti-inflammatory and anti-cancer properties [43,44,45]. Moreover, heparin has been widely used in bone regeneration since it can promote cell adhesion and osteogenic differentiation [46,47]. The NCC specimens modified with various amphiphilic polyanions were thoroughly characterized and used as fillers to prepare the PCL-based composites. Surface morphology, mechanical and *in vitro* biological properties were studied to evaluate the effect of NCC modification on filler distribution uniformity in the PCL matrix. Additionally, tensile characteristics, cell viability and osteoconductive properties of the composites were assessed. 

## 2. Materials and Methods

### 2.1. Chemicals and Supplements 

NCC (BGB Ultra^TM^, 8% *w*/*w* aqueous suspension) was purchased from Blue Goose Biorefineries Inc. (Saskatoon, SK, Canada). Heparin sodium salt from porcine mucosa (molecular weight 8000–25,000) was a product of AppliChem GmbH (Darmstadt, Germany). *N*-(Tert-butoxycarbonyl)-ethylenediamine (EDA(Boc), 97%) and *N_ε_*-Boc-*L*-lysine (Lys(Boc), 97%) were purchased from BLD Pharmatech Ltd. (Shanghai, China). 1-Ethyl-3-(3-dimethylaminopropyl)carbodiimide (EDC, ≥98%), *N*,*N′*-diisopropylcarbodiimide (DIC, 99%), 1-hydroxybenzotriazole hydrate (HOBT, ≥97%), (3-aminopropyl)triethoxysilane (APTES, ≥98%), 2-(*N*-morpholino)ethanesulfonic acid (MES, 99.5%) and *n*-hexylamine (98%) were purchased from Sigma-Aldrich (Darmstadt, Germany). *N*-Hydroxysuccinimide (NHS) and 2,4,6-trinitrobenzenesulfonic acid (TNBS, >98%) were supplied by Fluorochem Ltd. (Hadfield, UK) and Fluka (Buchs, Switzerland), respectively. Solvents and other reagents were supplied by Vecton Ltd. (St. Petersburg, Russia). All solvents were purified by distillation prior to use.

Amphiphilic P[Glu-*co*-Glu(OBzl)] (PGlu) used for NCC modification was synthesized as described earlier [40]. *γ*-Benzyl-*L*-glutamic acid *N*-carboxyanhydride and *n*-hexylamine were used as monomers and initiators. The molar ratio of monomer/initiator was 150. The number average molecular weight (*M_n_*), degree of polymerization and residual benzyl group (Bzl) content of PGlu according to ^1^H NMR were 8400, 56 and 21%, respectively. PCL, used as matrix polymer for composite materials, was synthesized using *ε*-caprolactone (monomer), tin(II) octoate (catalyst) and benzyl alcohol (initiator), as described earlier [41]. The weight average molecular weight (*M_w_*) and dispersibility (*Đ*) of the obtained PCL were determined by size-exclusion chromatography (SEC) using polystyrene standards and were 116,900 and 2.0, respectively. 

Dialysis membrane tubes Spectra/Pore (MWCO 1000, 3500) were purchased from Repligen Corp. (Boston, MA, USA).

### 2.2. Functionalization of Heparin

The Hep functionalization was performed in two steps. Initially, Hep covalent modification with an amino-bearing linker, namely, EDA(Boc), followed by removal of Boc-protecting groups was carried out using the activated ester method. In order to activate the Hep carboxyl groups, 30 mg (0.16 mmol) of EDC and 35 mg (0.30 mmol) of NHS were added to a solution of Hep (10 mL, 50 mg/mL) cooled to 2–4 °C in a 0.01 M MES buffer solution (pH 5.6). The reaction was carried out in an ice bath under constant stirring for 1 h. After that, 2 mL of a solution of EDA(Boc) (0.30 mmol) in 1,4-dioxane was added to the solution obtained in the previous step. The reaction was conducted at 30 °C under constant stirring for 24 h. Finally, the resulting solution was then transferred to a dialysis bag with a molecular weight cut-off (MWCO) of 3500 and purified by dialysis against distilled H_2_O for 1 week. The resulting product was freeze-dried. The yield of Hep–EDA(Boc) was 73%.

After that, the modification of Hep–EDA(Boc) with *n*-hexylamine (Hex–NH_2_) was also carried out using the activated ester method. In this case, 25 mg of EDC (0.13 mmol) and 27 mg of NHS (0.24 mmol) were added to a solution of Hep–EDA(Boc) (10 mL, 390 mg) in 0.01 M MES (pH 5.6) cooled to 2–4 °C. The reaction was continued for 1 h with constant stirring in an ice bath. Then, a 2 mL solution of Hex–NH_2_ (0.20 mmol) in 1,4-dioxane was added to the resulting solution of the activated Hep–EDA(Boc). The reaction was carried out at 30 °C for 24 h with constant stirring. Purification of Hep–(EDA(Boc), Hex) was performed in the same manner as described above for Hep–EDA(Boc). The yield of the final product was 95%.

Finally, the removal of Boc-protective groups was carried out. For this, 350 mg of functionalized heparin was dissolved in 9 mL of 4 M HCl solution in dry 1,4-dioxane and incubated at 22 °C with stirring for 1 h. The product was purified by dialysis against distilled H_2_O for 48 h using a dialysis bag with MWCO 3500. After that, the final product was freeze-dried. The yield of Hep modified with EDA and Hex (Hep-mod) was 68%.

Hep and Hep-mod samples were analyzed *via* SEC using a PolySep-SEC GFC-P 4000 column (7.8 × 300 mm, 5 μm, Phenomenex, Torrance, CA, USA) and Security Guard pre-column (7.5 × 35 mm, complete with GFC-4000, Phenomenex, Torrance, CA, USA). Analysis was performed at 40 °C, using a 0.1 M NaHCO_3_ solution as the mobile phase at a flow rate of 0.5 mL/min with refractometric detection. Dextrans with molecular weights of 4400–66,700 (Sigma-Aldrich, Darmstadt, Germany) were used as standards. The analysis showed that the *M_n_* and *Đ* of Hep-mod were 14,700 and 1.6, respectively.

### 2.3. Functionalization of Hydrophobized Poly(Glutamic Acid)

P[Glu-*co*-Glu(OBzl)] modification with lysine (Lys) was carried out *via* the interaction of activated carboxyl groups of PGlu with amino groups of Boc-lysine (Lys(Boc)) with the subsequent removal of Boc-protective groups. To activate 20 mol% carboxyl groups of PGlu, DIC (51 μL, 0.33 mmol) and HOBT (92 mg, 0.66 mmol) were added to 10 mL of P[Glu-*co*-Glu(OBzl)] solution (300 mg, 1.65 mmol carboxyl groups) in *N,N*-dimethylformamide (DMF) and kept at 22 °C under stirring for 20 min. A 5 mL Lys(Boc) solution (168 mg, 0.66 mmol) in water/acetonitrile (1/1, *v/v*) was added to the solution of activated PGlu and the reaction was carried out for 3 h at 22 °C under stirring. The product was purified by dialysis against water for 48 h using a dialysis bag with MWCO 3500 and freeze-dried. 

Finally, the removal of Boc-protection was performed by dissolving 110 mg of P[Glu-*co*-Glu(OBzl)] in 2 mL of 4 M HCl in dry 1,4-dioxane and incubating at 22 °C under constant stirring for 2 h. The resulting solution was transferred to a dialysis bag with MWCO 3500 and purified against distilled water for 48 h. The product was lyophilized. The yield of P[Glu-*co*-Glu(Lys)-*co*-Glu(OBzl)] (PGlu-Lys) was 75%.

### 2.4. Modification of NCC with Functionalized Heparin 

To attach Hep-mod to NCC, the latter was initially oxidized to generate aldehyde groups reactive toward the amino groups of Hep-mod. Oxidation was carried out according to the known protocol [48]. Briefly, 15 mL of water was added to 25 g of a commercial aqueous suspension of NCC (8% *w/w*, 10 mmol per glucose) and thoroughly stirred. After that, the mixture was cooled to 2–4 °C and 40 mL of an aqueous solution of sodium periodate (1.5 g, 7 mmol) was added. The reaction was run at 4 °C in the dark for 24 h. The resulting suspension was purified by dialysis against distilled water using dialysis bags with MWCO 1000. The resulting suspension was used for further reactions.

Modification of oxidized NCC with Hep-mod was performed as follows. A 10 mL solution of Hep-mod in 0.2 M borate buffer (BB, pH 8.5) was added to 25 mL of oxidized NCC suspension containing 500 mg of oxidized NCC. The used ratios of oxidized NCC/Hep-mod were equal to 1/0.25 (NCC–Hep-mod1) and 1/0.75 (NCC–Hep-mod2). The reaction was carried out under stirring at 22 °C for 4 h. The aldimine bonds formed between the amino groups of Hep-mod and the aldehyde groups of oxidized NCC, as well as the unreacted aldehyde groups of NCC, were reduced with sodium borohydride. For this purpose, 2 mL of aqueous NaBH_4_ solution (4 mg) was added to the resulting reaction system and incubated for 2 h. Unreacted Hep-mod was removed by several cycles of centrifugation of the suspension (20 °C, 8000 rpm, 10 min), the supernatant was taken up and the precipitate was washed with a fresh portion of water. This procedure was repeated three times. All washing solutions were combined and lyophilized. The yields of NCC–Hep1 and NCC–Hep2 were about 50%.

### 2.5. Modification of NCC with Hydrophobized Poly(Glutamic Acid) 

This approach included the initial silanization of NCC with 3-aminopropyltriethoxysilane (APTES) followed by P[Glu-*co*-Glu(OBzl)] attachment *via* the reaction between the amino groups of APTES and the activated carboxyl groups of P[Glu-*co*-Glu(OBzl)]. Silanization was carried out as described elsewhere [49,50]. For this purpose, 10 mL of ethanol was added to 9 g of a commercial aqueous suspension of NCC (8% *w/w*), centrifuged (20 °C, 8000 rpm, 10 min), and the supernatant was completely removed. The wet precipitate was placed into 60 mL of an ethanol/water solution (3/1, *v/v*) acidified with glacial acetic acid to pH 4.2. The resulting suspension was dispersed using an ultrasound bath and kept overnight at 4 °C. A previously prepared solution of hydrolyzed APTES was added dropwise to the suspension under vigorous stirring (4 mL of APTES mixed with 20 mL of an ethanol/water mixture acidified with glacial acetic acid to pH 4.2 for 30 min). The reaction was carried out at 22 °C with constant stirring for 3 h. The obtained product was washed by centrifugation (20 °C, 8000 rpm, 10 min) with an ethanol/water mixture 8 times. The resulting precipitate was cured at 105 °C for 15 min in a desiccator. The yield of silanized NCC (NCC-APTES) was 28%.

After that, modification of NCC–APTES with P[Glu-*co*-Glu(OBzl)] was carried out. In the first step, the carboxylic groups were activated by adding DIC (57 μL, 0.37 mmol-COOH groups) and HOBT (102 mg, 0.73 mmol) to a 10 mL solution of P[Glu-*co*-Glu(OBzl)] (200 mg, 1.22 mmol-COOH groups) in DMF. The amounts of DIC and HOBT were calculated to activate 30 mol% carboxyl groups of P[Glu-*co*-Glu(OBzl)]. The activation reaction was carried out for 20 min at 22 °C under constant stirring. The P[Glu-*co*-Glu(OBzl)] solution was mixed with 10 mL of an aqueous suspension containing 267 mg of NCC–APTES in mass ratios of NCC–APTES/P[Glu-*co*-Glu(OBzl)] = 1/0.25 or 1/0.75 (samples NCC–APTES–PGlu1 or NCC–APTES–PGlu2, respectively). The reaction was carried out at 22 °C under constant stirring for 3 h. The resulting NCC–APTES–PGlu was washed by centrifugation (20 °C, 8000 rpm, 10 min) ten times with DMF. All collected washing solutions were combined and purified by dialysis against water (MWCO 1000) for 48 h. The products were dried *via* lyophilization. The yields of NCC–APTES–PGlu1 and NCC–APTES–PGlu2 were 50–52%.

### 2.6. Modification of NCC with Lysine-Functionalized Poly(Glutamic Acid) 

Modification of pre-oxidized NCC (see Section 2.4) with PGlu–Lys was carried out as follows. A 10 mL PGlu–Lys solution (51 mg) in 0.2 M BB (pH 8.5) was added to 10 mL of aqueous suspension containing 204 mg of oxidized NCC. The reaction was carried out at room temperature under stirring for 4 h. Thereafter, the reduction of residual aldehyde groups and aldimine bonds formed was performed by adding 2 mL of aqueous NaBH_4_ solution (4 mg) to the reaction system. The reaction was left to run at 22 °C under stirring for 2 h. The mass ratio of oxidized NCC/PGlu–Lys was 1/0.25. The resulting product was washed by centrifugation (25 °C, 8000 rpm, 10 min) five times with water and then ten times with dimethyl sulfoxide (DMSO). All washing solutions were combined, purified by dialysis against water for 8 h using dialysis bags (MWCO 1000) and freeze-dried. The yield of NCC–PGlu–Lys was 49%. 

### 2.7. Characterization of Functionalized NCC

The amount of polyanions bound to NCC was determined as the difference between the initial mass of polyanion taken for the reaction and the mass of unbound polyanion separated after the reaction. The amount of unreacted Hep-mod was determined using a UV-Vis SF-56 spectrophotometer (SPEKTR LLC, St. Petersburg, Russia). For this purpose, separated, purified and dry samples of unbound Hep-mod were dissolved in 10 mL of water and filtered through a syringe nylon filter with a pore size of 0.22 μm. Then, 0.1 mL of the resulting solution was withdrawn and diluted in 0.9 mL of water. An aliquot of this solution (0.5 mL) was added to 1.5 mL of borate buffer (0.0125 M, pH 9.4) and 0.1 mL of aqueous TNBS solution (8 mg/mL) was further added to the above solution. Subsequently, the solution was thoroughly mixed for 30–60 s using a Vortex V-1 plus vortex shaker (Biosan, Riga, Latvia) and then incubated at room temperature (RT) for 50 min. At the end of the reaction, 1.5 mL of citrate buffer solution (0.01 M, pH 4.8) was added to the analyzed solution and mixed thoroughly on the vortex shaker for 60 s. The optical density was measured at 422 nm. The amount of Hep-mod in the solution was determined from the calibration plot built for standard solutions of Hep-mod in the concentration range of 0.15–1.40 mg/mL.

The amount of P[Glu-*co*-Glu(OBzl)] and P[Glu-*co*-Glu(Lys)-*co*-Glu(OBzl)] bound to NCC was also estimated by spectrophotometric analysis of their content in the washes collected after NCC modification. In both cases, the analysis was performed at 265 nm and calculations were performed regarding calibration plots of the corresponding copolymers. 

Fourier-transform infrared spectroscopy (FTIR) was performed using the IRAffinity-1S instrument (Shimadzu Corp., Kyoto, Japan). Samples of 0.2 mg in 20 mg of KBr were scanned in the diapason of 400–4000 cm^−1^ with a scan number and resolution equal to 40 and 2 cm^−1^, respectively. 

^1^H and ^13^C NMR spectroscopy for the obtained substances was performed using AVANCE AV-400 and AVANCE II 500 (Bruker, Karlsruhe, Germany), respectively. The spectra were processed using the TopSpin 2.1 software (Bruker BioSpin, Rheinstetten, Germany). To register ^1^H NMR spectra, DMF-d7, DMSO-d6 and D_2_O were used as solvents for PGlu, PGlu–Lys and Hep and Hep-mod, respectively.

The hydrodynamic diameter (*D_H_*) and electrokinetic potential (ζ-potential) of NCC before and after modification were measured with a Zetasizer Nano-ZS (Malvern Panalytical, Malvern, UK) equipped with a He–Ne laser (633 nm). A suspension of NCC/modified NCC was prepared using bi-distilled water and water alkalized to pH 7.5 with NaOH under short-term ultrasonication (10 s) using a Sonopuls MS 73 ultrasound probe (Bandelin, Berlin, Germany) at a power of 40%. Measurements were performed at 25 °C and a scattering angle of 173°.

Thermogravimetric analysis (TGA) was performed in an air atmosphere using a DTG-60 thermoanalytical instrument (Shimadzu, Tokyo, Japan). The heating rate and temperature range were 5 °C/min and 100–700 °C, respectively.

### 2.8. Fabrication of Composite Films

A detailed description of the preparation of PCL-based composite films can be found in our previous paper [41]. Briefly, composite films containing 10 wt% of filler were obtained by casting 6.8 mL of filler suspension in PCL solution onto the cellophane surface. For this purpose, the water-soaked cellophane was previously fixed on a glass ring (i.d. 76 mm, height 30 mm) and dried for 12 h at room temperature. Chloroform or chloroform/acetonitrile mixture (1:1, *v*/*v*) was used as a solvent for PCL. The casted film was dried for 12–14 h at room temperature on a cellophane substrate and then transferred onto a glass substrate and dried in the thermostat at 40 °C for 2 weeks.

### 2.9. Characterization of Composites 

#### 2.9.1. Morphology 

The morphology of the obtained composite films based on PCL and modified NCC was investigated and compared with unfilled PCL and PCL/NCC *via* optical microscopy in transmitted and reflected light using an Eclipse E200 microscope (Nikon, Tokyo, Japan), as well as *via* scanning electron microscopy (SEM) using a JSM-7001F Jeol microscope (Tokyo, Japan). Prior to SEM analysis, gold was deposited on the surface of the film specimens. 

#### 2.9.2. Tensile Properties 

The mechanical properties of the polymer films were determined *via* uniaxial tensile testing using an AG-100X Plus universal tensile tester (Shimadzu Corp., Kyoto, Japan) at room temperature, according to ASTM D0638 requirements [50]. The tested specimens had the following dimensions: length = 20 mm, width = 2 mm and thickness = 83 ± 10 μm, respectively. The tensile speed was 10 mm/min.

### 2.10. In Vitro Biological Evaluation 

Osteoblast-like cells (human osteosarcoma MG-63 cell line) and human fetal mesenchymal stem cells (FetMSCs) were used for the cytotoxicity studies. Both cell lines were purchased from the Cell Collection of Institute of Cytology of Russian Academy of Sciences (St. Petersburg, Russia). MG-63 cells were cultured in a CO_2_ incubator at 37 °C in a humidified atmosphere containing air and 5% CO_2_ in MEM cell culture medium (modified Eagl’s medium; Gibco, Billings, MT, USA) containing 1% essential amino acids, 10% (*v/v*) heat-inactivated fetal bovine serum (FBS; HyClone, Logan, UT, USA), 1% *L*-glutamine, 50 U/mL penicillin and 50 μg/mL streptomycin. FetMSCs were cultured in a CO_2_ incubator at 37 °C in a humidified atmosphere containing air and 5% CO_2_ in DMEM/F12 cell culture medium (Dulbecco’s modified Eagl’s medium; Gibco, Billings, MT, USA), containing 10% (*v/v*) heat-inactivated fetal bovine serum (FBS; HyClone, Logan, UT, USA), 1% *L*-glutamine, 50 U/mL penicillin, and 50 μg/mL of streptomycin. 

#### 2.10.1. Cell Viability 

The study was performed in non-adhesive 96-well plates. Initially, the round-shaped film specimens with a diameter of 6 mm were stuck to the bottom of the wells using BF-6 medical glue (Tula Pharmaceutical Factory, Tula, Russia). The specimens were sterilized *via* UV exposure for 15 min. The plastic surface of the adhesive 96-well cell culture plate was used as a control surface (positive control). Then, 5 × 10^3^ cells/100 µL/well were seeded onto the film specimens in 96-well plates. The cells were cultured for 72 h. At the end of the incubation period, the medium was removed and 50 μL/well of DMEM/F12 (FetMSCs) or MEM (MG-63) media containing an MTT reagent (0.1 mg/mL) was added. Cells were incubated in a CO_2_ incubator for 2 h at 37 °C. After removal of the supernatant, the formazan crystals formed by the metabolically viable cells were dissolved in DMSO (50 µL/well). Then, the solutions were transferred to clean wells, and the optical density at 570 nm was measured using a Thermo Scientific Multiskan flatbed spectrophotometer (Thermo Fisher Scientific, Waltham, MA, USA). 

#### 2.10.2. Osteogenic Differentiation 

FetMSCs (20 × 10^3^ cells/cm^2^) were seeded onto the surface of the film specimens glued to the bottom of the wells of a 96-well plate and cultured to achieve a monolayer (3 days until confluence 90%). Then, the medium was changed to MSCgo differentiation medium (Biological Industries, Beit-Haemek, Israel) containing 10% (*v/v*) thermal-inactivated FBS (HyClone, Logan, UT, USA), 1% *L*-glutamine, 50 U/mL penicillin, 50 μg/mL streptomycin, *β*-glycerophosphate, dexamethasone (1000×) and ascorbic acid (Sigma-Aldrich, Darmstadt, Germany). 

*ALP detection.* After 14 days of culturing, the specimens were washed three times with 1x PBS and treated with 4% neutral formalin for 30 min to fix the cells. The specimens were washed with PBS three times for 5 min and then stained with a BCIP–NBT mixture (5-bromo-4-chloro-indolyl phosphate tetrazole blue, Sigma, Darmstadt, Germany) for 30 min in the dark. The images were captured *via* optical microscopy in transmitted light using the Eclipse e200 microscope equipped with a U3CMOS digital camera (×4) and NIS-Elements F software (version 3.0, Nikon, Tokyo, Japan).

*Biomineralization study.* After 28 days of cell culturing on the surface of the films, the specimens were washed and processed to fix the cells as described for ALP detection. After that, one set of the film specimens was stained with an alizarin red S dye. In this case, 100 µL of dye dissolved in deionized water (20 mg/mL) was added to each well containing a film specimen. All specimens were incubated at room temperature for 40 min. After that, the specimens were washed with water until the coloration of the washing water disappeared. The images were registered by optical microscopy in transmitted light using the Eclipse e200 microscope equipped with a U3CMOS digital camera (×4) and NIS-Elements software (Nikon, Tokyo, Japan). Another set of film specimens was dyed with calcein by adding 100 μL of dye dissolved in deionized water (5 μg/mL) to each well containing a testing specimen. The plates were incubated at 4 °C for approximately 14 h. The specimens were then washed with water 12–15 times. Calcein fluorescence was measured using a Mikmed-2 (Lomo, St.-Petersburg, Russia) fluorescence microscope at an emission wavelength of 530 nm.

### 2.11. Statistics 

Quantitative analysis of the polyanions bound to the NCC was performed in a series of three independent measurements. Seven to thirteen specimens of each type of film were tested for their tensile properties, and the data were averaged. All biological experiments were performed in at least three independent repeats. Quantitative data were processed using the Excel MS program and presented as mean ± standard deviation. 

## 3. Results and Discussion

### 3.1. Chemical Modification and Characterization of NCC

**Figure 1:**
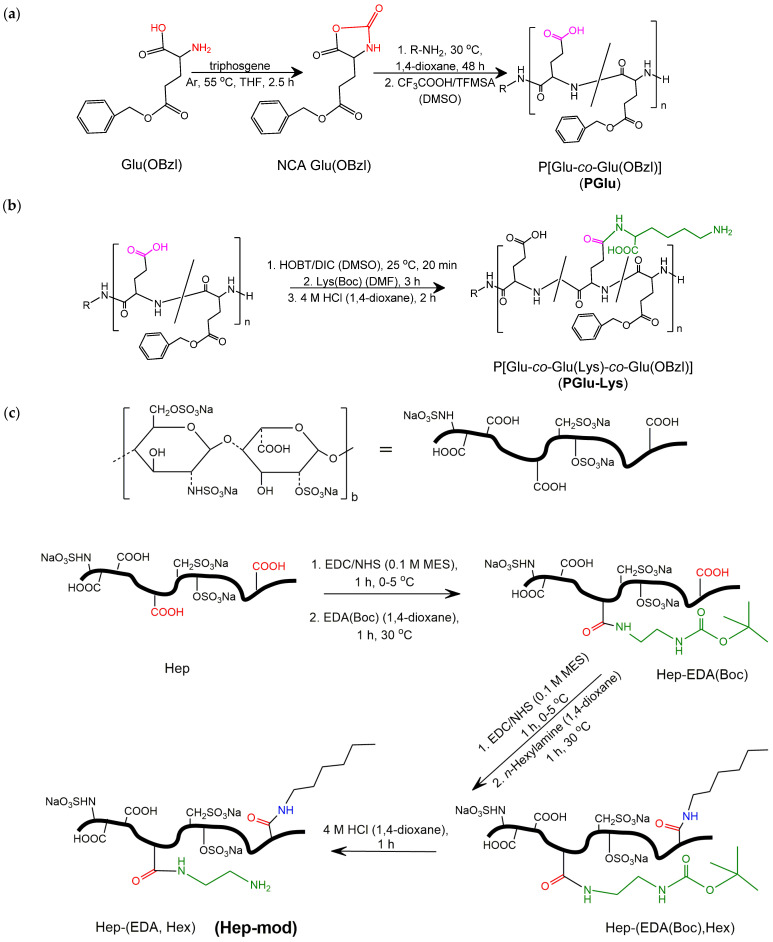
Scheme of functionalized polyanion synthesis for further chemical modification of NCC: (**a**) synthesis P[Glu-*co*-Glu(OBzl)] (PGlu) *via* ring-opening polymerization of *N_α_*-carboxyanhydride of Glu(OBzl) (Glu(OBzl) NCA); (**b**) synthesis of P[Glu-*co*-Glu(Lys)-*co*-Glu(OBzl)] (PGlu-Lys) by post-polymerization modification of P[Glu-*co*-Glu(OBzl)] with *L*-lysine; and (**c**) modification of heparin with ethylenediamine and *n*-hexylamine (Hep-mod). Abbreviations: THF—tetrahydrofuran, TFMSA—trifluoromethanesulfonic acid.

Heparin and poly(glutamic acid) derivatives were selected as bioactive polyanions for the chemical modification of NCC and further preparation of biocomposites suitable for bone regeneration. The selection of polyanions was motivated by the necessity to impart the osteoconductive properties to the PCL/NCC composites. However, both Hep and PGlu in their neat form are hydrophilic polymers, which could barely improve the distribution of hydrophilic NCC in a matrix of hydrophobic PCL. Considering this, hydrophobized derivatives of Hep and PGlu were preliminarily synthesized and further modified with amine-bearing linkers. The scheme of polyanion functionalization is presented in Figure 1. The synthesized amphiphilic polyanions were characterized for their composition using ^1^H NMR spectroscopy (Figure S1, Supplementary Materials). The molecular weight characteristics and composition of the functionalized polyanions are summarized in Table 1. 

**Figure 2:**
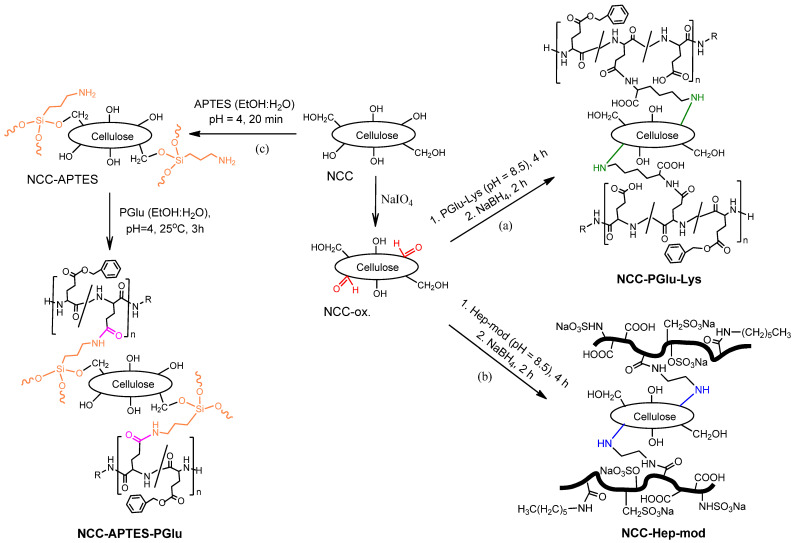
Scheme of NCC modification with amphiphilic polyanions: NCC oxidation with sodium periodate and modification with (**a**) PGlu–Lys and (**b**) Hep-mod, respectively, and (**c**) silanization with APTES followed by reaction with activated PGlu.

Figure 2 illustrates the pathways used for the modification of NCC with functionalized polyanions. Two of the used approaches included the pre-oxidation of the surface vicinal diols of glucose in NCC to generate highly reactive aldehyde groups capable of reacting with the amino groups of Hep-mod and PGlu–Lys. The third approach was based on the silanization of NCC with APTES, followed by the reaction of the introduced amino groups with activated carboxyl groups of PGlu. 

The modification of NCC with PGlu–Lys was carried out at a mass ratio of NCC/PGlu–Lys = 1:0.25, while the modification of NCC with PGlu and Hep-mod was performed at mass ratios of NCC/polyanion = 1:0.25 and 1:0.75. Based on the spectrophotometric analysis of unreacted amounts of polymers, the content of immobilized Hep-mod was 153 ± 10 mg/g NCC (specimen NCC–Hep-mod1) and 390 ± 22 mg/g NCC (specimen NCC–Hep-mod2) for initial mass ratios of NCC/Hep-mod of 1:0.25 and 1:0.75, respectively. The content of immobilized PGlu was found to be 47 ± 4 mg/g NCC (specimen NCC–APTES–PGlu1) and 526 ± 42 mg/g NCC (specimen NCC–APTES–PGlu2) at initial component mass ratios of 1:0.25 and 1:0.75, respectively. The content of immobilized PGlu–Lys was 127 ± 9 mg/g NCC (specimen NCC–PGlu–Lys).

**Figure 3:**
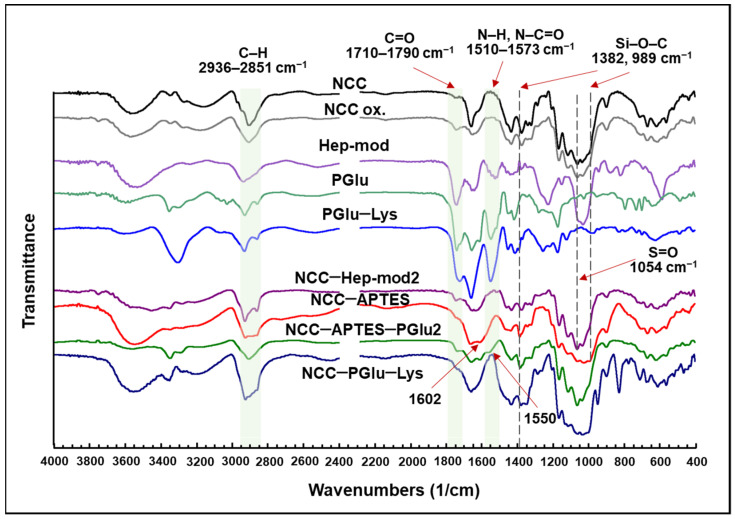
FTIR spectra of the neat NCC and NCC modified with various polyanions.

FTIR spectra of the neat and modified NCC are presented in Figure 3. For all modified NCC specimens, including NCC–APTES, the appearance or intensification of characteristic bands at 2936–2851 cm^−1^, corresponding to symmetric and asymmetric C–H stretching vibrations of alkyl fragments, was observed [51,52]. This band indicates the coupling of APTES and polyanions to NCC. For all polyanion-modified NCCs compared to unmodified NCCs, an increase in band intensity in the 1710–1790 cm^−1^ area (namely 1732 or 1737 cm^−1^) corresponding to C=O stretching vibrations of -COOH groups of Hep [52] or PGlu/PGlu–Lys [53] was also observed. In the case of oxidized NCC (NCC-ox.), an increase in the intensity of the band at 1737 cm^−1^ corresponding to C=O stretching vibrations of the formed aldehyde groups was detected. Furthermore, the appearance or increase in band intensity was observed in the 1573–1510 cm^−1^ area for NCC–Hep-mod and NCC–PGlu–Lys due to the N–H bending and N–C=O stretching vibrations of amide II (for PGlu, PGlu–Lys and Hep) [52,54] and the N–H bending vibrations of primary and acetylated (NHAc) Hep amino groups [52]. In the case of NCC–Hep, an increase in the intensity of the band at 1054 cm^−1^, corresponding to asymmetrical stretching of the S=O fragment of –CH_2_OSO_3_, indicates the successful modification of NCC with heparin [53]. The successful modification of NCC with APTES is also supported by the increase in the band intensity at 1602 cm^−1^ and a broadening to about 1519 cm^−1^, corresponding to the N–H bending vibration of the primary amino groups of APTES [49]. After NCC–APTES modification with PGlu, the appearance of a characteristic band at 1550 cm^−1^ was observed, which can be attributed to N–H bending vibrations of PGlu amide groups and residual (unreacted) primary amino groups of APTES [49,52,54]. For NCC–APTES and NCC–APTES–PGlu, FTIR spectra show the appearance of a new band at 1382 cm^−1^ and an increase in the intensity of the band at 989 cm^−1^ in the spectrum of NCC–APTES. Both of these signals can be related to the symmetrical stretching of the Si–O–C bond [55]. 

The obtained samples of modified NCC were investigated by solid-state ^13^C NMR spectroscopy. Examples of spectra are shown in Figure S2 (Supplementary Materials). Since heparin and NCC are both polysaccharides, it is difficult to see differences in the ^13^C NMR spectra of NCC and NCC–Hep-mod. Nevertheless, for NCC–Hep-mod, the appearance of signals at 63 and 97 ppm can be attributed to the carbon atoms of the methylene and methine groups of Hep (Figure S2a, Supplementary Materials). 

The ^13^C NMR spectrum of NCC–APTES contained signals at 5, 17 and 37 ppm, which correspond to the carbon atoms of the methylene groups of APTES [51]. In the case of NCC–APTES–PGlu, a number of signals corresponding to both APTES and PGlu were observed: the signals at 6 and 18 ppm corresponded to the carbon atoms of the methylene group of APTES; the signal at 33 ppm corresponded to the carbon atom of the methylene group of hexylamine included in PGlu; the signal at 45 ppm corresponded to the carbon atom of the methylene group of APTES and of hexylamine included in PGlu; and the signals at 54, 127 and 138, as well as 174 ppm were the signals of the carbon atom of the methine group of PGlu, the carbon atom of the aromatic ring of PGlu, and the carbon atoms of the carbonyl and carboxylate atoms of PGlu, respectively [40,51] (Figure S2b, Supplementary Materials). 

The ^13^C NMR spectrum of NCC–PGlu–Lys showed signals corresponding to the methylene group carbon atom of Lys (16 ppm), the methylene group carbon atoms of Lys and PGlu (28 and 40 ppm, respectively), the aromatic ring carbon atom of PGlu (129 and 139 ppm), the carbonyl and carboxylate carbon atoms of Lys and PGlu (176 ppm) [55,56,57] (Figure S2c, Supplementary Materials). Overall, the results of FTIR and solid-state NMR spectroscopy indicated that the NCC modification was successful in all cases.

**Figure 4:**
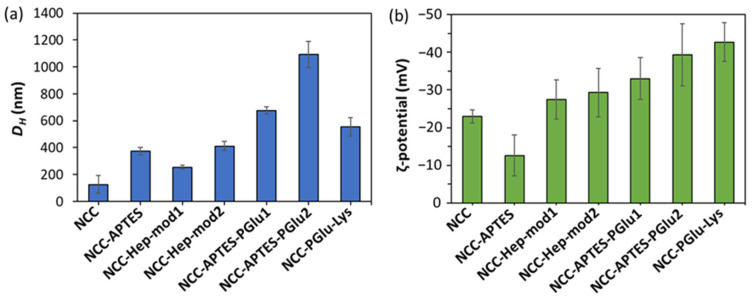
Physicochemical characteristics of neat and modified NCC specimens: (**a**) hydrodynamic diameter; (**b**) electrokinetic potential (ζ-potential).

In addition, the specimens of neat and modified NCC were analyzed by dynamic and electrophoretic light scattering to monitor changes in NCC characteristics as a result of modification (Figure 4). It can be seen that modification of NCC with APTES contributed to the increase in the hydrodynamic diameter of NCC particles (Figure 4a) and ζ-potential (Figure 4b). Subsequent modification of NCC–APTES with PGlu led to a further increase in the size of these particles, but was accompanied by a decrease in ζ-potential due to the appearance of PGlu carboxyl groups on the surface. Similarly, modification of NCCs with Hep-mod and PGlu–Lys resulted in a decrease in surface ζ-potential and an increase in hydrodynamic diameter, but not as significant as in the case of the intermediate modification with APTES. Thus, the changes in NCC characteristics also indirectly indicate the success of NCC modification. 

**Figure 5:**
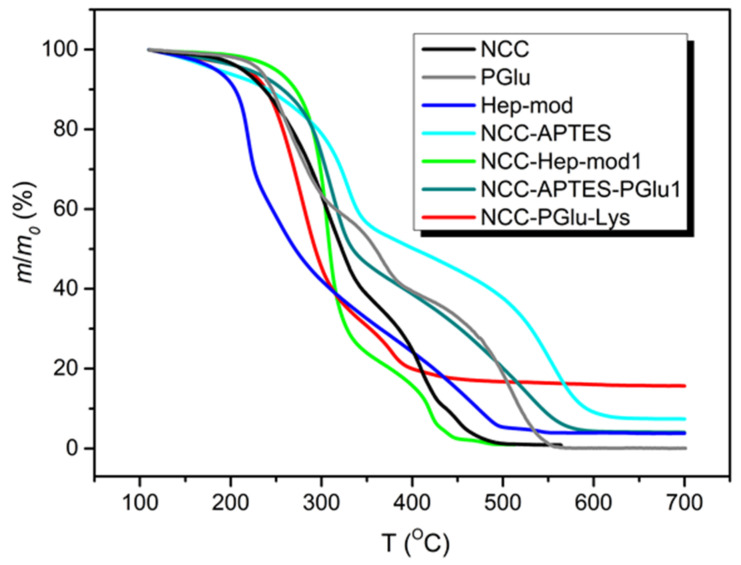
TGA curves of PGlu and Hep-mod used for NCC modification, as well as neat and modified NCC.

The modified NCC specimens and individual components were investigated by TGA. Examples of TGA curves as well as the obtained values of *τ*_5_ and *τ*_10_ reflecting the degradation temperatures of 5 and 10% modified NCC, respectively, are shown in Figure 5 and Table S1 (Supplementary Materials). The thermal destruction of heparin modified with hexylamine and ethylenediamine exhibited a complex pattern. A slow mass reduction of about 4% was observed up to 160 °C. Further heating demonstrated a stage of accelerated mass decrease up to 230 °C (~28% mass drop), followed by a slow mass loss between 230–260 °C. In the 260–410 °C interval, the mass reduction process was further slowed down and the mass dropped to 22%. In the final step (the 410–495 °C region), a decrease in mass was found from 22 to 6%. In general, similar thermal degradation profiles were observed for NCC and heparin-modified NCC. The TGA curve of neat NCC demonstrated three temperature intervals of NCC mass decrease: up to 350 °C, with a mass loss of 61%; in the range of 350–430 °C, with a mass loss of 27%; and up to 500 °C, with a mass loss of 11%. Finally, the mass loss was reduced to about 1% of residual mass. In the case of NCC–Hep-mod, similar ranges of mass drop temperatures were observed, but unlike NCC, two intervals were clearly distinguished in the last stage of thermal degradation, indicating the modification of NCC by heparin. Namely for NCC–Hep-mod in the temperature range of 200–320 °C, an intensive mass decrease was observed (64% mass loss). Then, a slow mass decrease of 22 and 10% was observed when heated in the intervals of 320–410 and 410–440 °C, respectively. A subsequent increase in temperature to 490 °C was accompanied by a slow decrease of mass to about 1% and stabilization of this residual mass.

The shapes of the TGA curves of NCC–APTES and NCC–APTES–PGlu were generally similar to each other, except for the boundaries of some mass loss temperature ranges, and were significantly different from the TGA curve of unmodified NCC. The NCC–APTES specimen exhibited the following mass loss steps: a mass loss of 6% up to 200 °C, an intensive mass reduction by 37% between 200–350 °C, a slow mass reduction by 16% between 350–480 °C, an intensive mass drop by 32% in the range of 480–600 °C, and finally, a slow mass drop to a residual mass of ~7% when heated to 650 °C. In the case of NCC–APTES–PGlu, a monotonic mass decrease of 4% occurred at an approximately constant rate when heated to 190 °C. Thereafter, a more pronounced mass loss was observed compared to NCC–APTES. Specifically, a 45% mass drop was detected from 190 to 330 °C, and a 17% mass reduction was achieved between 330 and 425 °C. The second intensive stage of mass decrease of 30% was observed in the interval of 425–580 °C, after which the mass of the sample almost immediately stabilized at the level of 4%. The change of the temperature range boundaries for NCC–APTES–PGlu, as well as the increase in the degree of degradation of this specimen in the interval 190–320 °C was obviously caused by the addition of PGlu. The TGA curve of free PGlu showed two stages of intensive mass decrease in the ranges of 190–320 °C and 400–560 °C, by 40 and 39%, respectively, whereas a slower mass decrease (of 18%) was observed from 330 to 400 °C.

In the case of NCC–PGlu–Lys, the TGA curve differed from the TGA curve of NCC. The boundaries of thermal destruction stages were more similar to PGlu, indicating the attachment of the latter to NCC. In particular, a slow mass drop of ~3% was observed when NCC–PGlu–Lys was heated in the range of 120–195 °C. After that, the main stage of accelerated thermal degradation, with a mass decrease of 54%, was detected between 210 and 310 °C. Further heating to 390 °C was followed by a second stage of accelerated mass drop of 23%. Then the mass drop slowed down and stabilized to about 15% at 620 °C.

Cumulatively, NCC modification with Hep-mod and PGlu resulted in increased *τ*_5_ and *τ*_10_ values. Overall, *τ*_5_ and *τ*_10__v_ values for modified NCC specimens ranged from 216–250 and 239–273 °C, respectively. These results indicate the possibility of using modified NCC as a filler to produce 3D composite materials using additive technologies such as fused deposition modeling (FDM 3D-printing technique). Such thermoplastic polymers as PCL (melting point 55–70 °C), polylactides (melting point 150–200 °C), poly(3-hydroxybutyrate) (melting point 165–185 °C) and other biodegradable polyesters with melting points up to 200 °C [26] can be used as a polymer matrix for production of 3D-printed composites. 

### 3.2. Manufacturing and Characterization of Composite Films

The upper limit of filler content acceptable and appropriate for introduction into the polymer matrix depends on its properties and ability to provide homogeneous distribution and minimal aggregation, affecting the mechanical characteristics. It was previously found that an increase from 5 to 15 wt% in the content of pure NCC or NCC modified with a short glutamic acid peptide (polymerization degree = 6) was accompanied by a marked decrease in the mechanical properties of PLA-based composites when 15 wt% of filler was used [39,41]. At the same time, no change in biological properties was observed when using 10 and 15 wt% of filler in the composite. Thus, in this study, 10 wt% of neat or modified NCC as a filler was used to prepare the PCL-based composite films.

**Figure 6:**
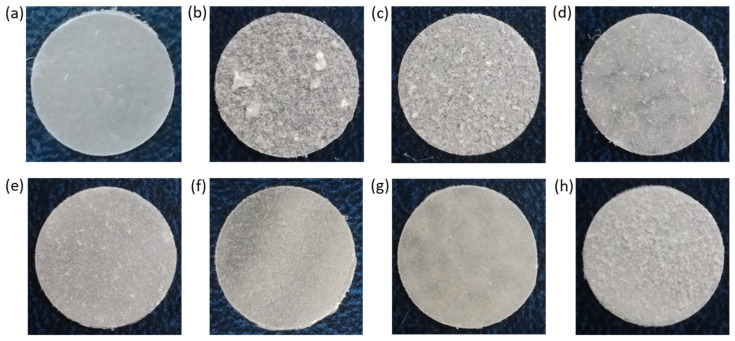
Photographs of PCL and PCL-based composite films with neat and modified NCC: (**a**) PCL (CHCl_3_); (**b**) PCL/NCC (CHCl_3_); (**c**) PCL/NCC–Hep-mod1 (CHCl_3_); (**d**) PCL/NCC–Hep-mod1 (AcN/CHCl_3_); (**e**) PCL/NCC–Hep-mod2 (AcN/CHCl_3_); (**f**) PCL/NCC–APTES–PGlu1 (AcN/CHCl_3_); (**g**) PCL/NCC–APTES–PGlu2 (AcN/CHCl_3_); and (**h**) PCL/NCC–PGlu–Lys (AcN/CHCl_3_). Filler content of 10 wt%; film diameter of 1 cm.

**Figure 7:**
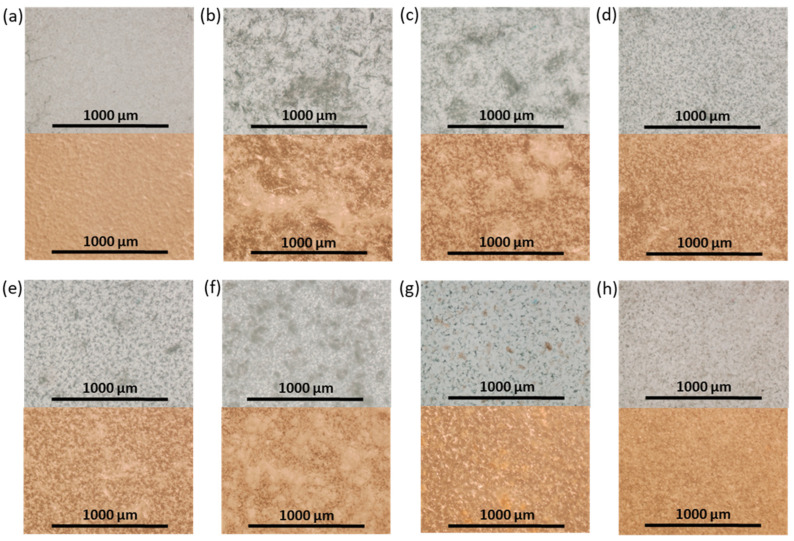
Optical transmitted light (top) and reflected (bottom) microscopy images of neat and composite PCL-based films obtained (×4): (**a**) PCL (CHCl_3_); (**b**) PCL/NCC (CHCl_3_); (**c**) PCL/NCC–Hep-mod1 (CHCl_3_); (**d**) PCL/NCC–Hep-mod1 (AcN/CHCl_3_); (**e**) PCL/NCC–Hep-mod2 (AcN/CHCl_3_); (**f**) PCL/NCC–APTES–PGlu1 (AcN/CHCl_3_); (**g**) PCL/NCC–APTES–PGlu2 (AcN/CHCl_3_); and (**h**) PCL/NCC–PGlu–Lys (AcN/CHCl_3_). Filler content of 10 wt%.

In order to produce the films, a suspension of NCC in 5% PCL solution in organic solvent was cast on a cellophane substrate and then dried. Figure 6 shows photographs of the obtained control PCL and composite films. Initially, CHCl_3_ was used as a solvent for the manufacturing of the PCL films and PCL-based composites. However, the formation of large agglomerates was observed both for neat and modified NCC particles when CHCl_3_ was used for the production of composite films (Figure 6b,c). The partial replacement of non-polar CHCl_3_ with polar AcN (CHCl_3_/AcN mixture (1/1, *v/v*)) led to a significant improvement in the distribution of the modified NCC in the PCL matrix (Figure 6d–h), while no evident effect was observed when unmodified NCC was used as a filler. These results are in agreement with the evaluation of the film morphology *via* optical microscopy (Figure 7). Comparison of the images in Figure 6c,d as well as the images in Figure 7c,d led to the conclusion that a more homogeneous distribution of NCC–Hep1 in the PCL matrix was achieved when the AcN/CHCl_3_ system was used to manufacture the composite film.

**Figure 8:**
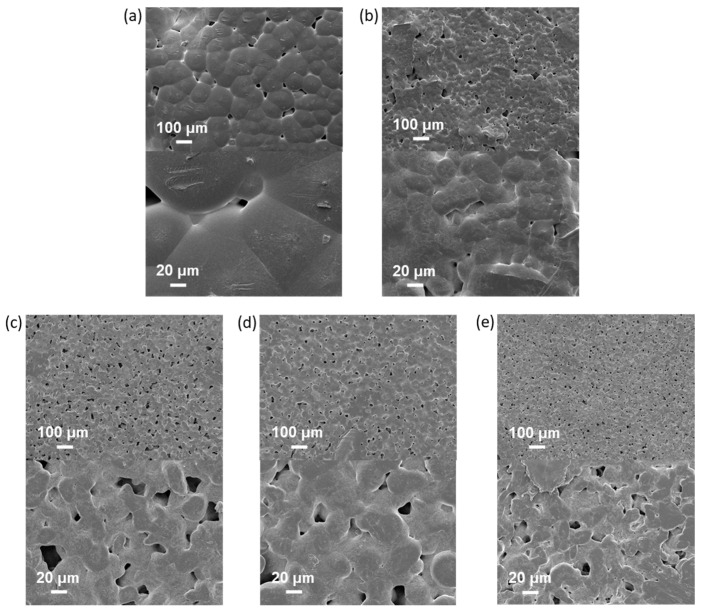
Scanning electron microscopy images of neat and composite PCL-based films obtained (top ×100, bottom ×100): (**a**) PCL (CHCl_3_); (**b**) PCL/NCC (AcN/CHCl_3_); (**c**) PCL/NCC–Hep-mod2 (AcN/CHCl_3_); (**d**) PCL/NCC–APTES-PGlu2 (AcN/CHCl_3_); and (**e**) PCL/NCC–PGlu–Lys (AcN/CHCl_3_). Filler content: 10 wt%.

SEM images showed a rough PCL/NCC surface due to the presence of large NCC agglomerates in the near-surface layer (Figure 8a). In turn, improved filler distribution in the polymer matrix was observed in the case of PCL-based composites filled with modified NCC (Figure 8b–e). In addition, an increase in porosity and a decrease in the size of polymer microglobules were also observed in all composed materials.

**Figure 9:**
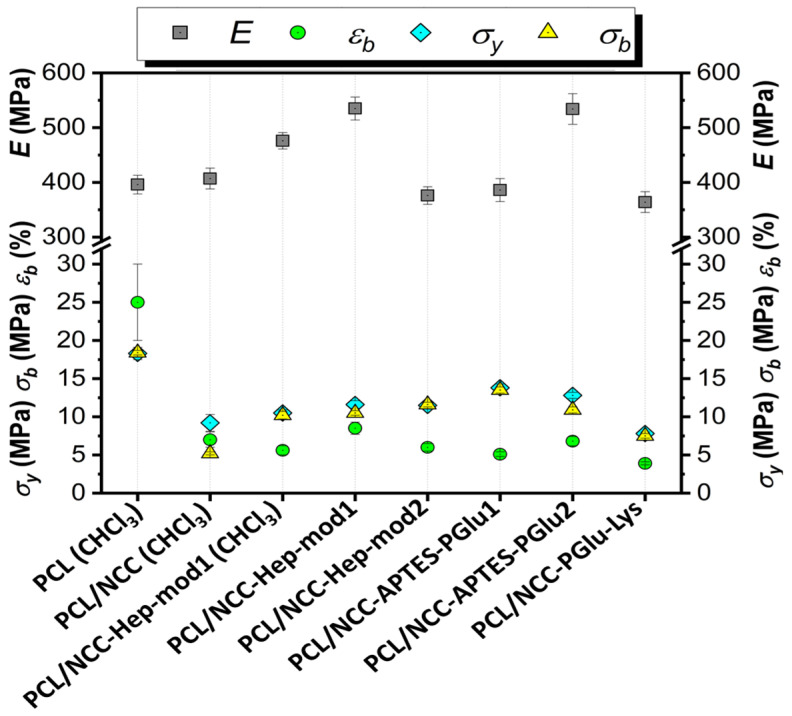
Tensile properties of PCL-based composite films filled with neat and modified NCC (filler content of 10 wt%, solvent system used for films casting is AcN/CHCl_3_, if not specified otherwise).

The mechanical characteristics of the composite films were evaluated by determining the following parameters in uniaxial tension: Young’s modulus (*E*), elongation at break (*ε_b_*), ultimate tensile strength (*σ_b_*) and yield strength (*σ_y_*). The results obtained are summarized in Figure 9. Some examples of stress-strain curves can be found in Figure S3 (Supplementary Materials).

The mechanical characteristics of the composite films show significant differences in how NCC affects the properties of the matrix polymer PCL. This effect is due to both the way the NCC is modified and the type of solvent used to make the polymer film (Figure 9). The use of CHCl_3_ as a solvent led to intense aggregation of neat NCC in the film volume (Figure 6). Aggregation of the filler creates zones of inhomogeneity in the film, where defects and ruptures are primarily formed during tensile strength. Therefore, composite films containing unmodified hydrophilic NCC particles as fillers and cast from CHCl_3_ were markedly inferior to the matrix PCL film in terms of overall mechanical performance. 

Testing a composite film cast from CHCl_3_ and filled with NCC modified with amphiphilic functionalized heparin (NCC–Hep-mod1) demonstrated a significant increase in the stiffness due to the NCC modification. The elastic modulus of the composite material, even with such a heterogeneous morphology, was 20% higher than that of the PCL film. In turn, the composite film, cast from an AcN/CHCl_3_ (1/1, *v/v*) mixture and having a more homogeneous filler distribution (Figure 6), comprised a 35% higher elastic modulus than the PCL film and a 12% higher elastic modulus than the PCL/NCC–Hep-mod1 film cast from CHCl_3_.

The introduction of NCC–Hep-mod2, which contains a larger amount of immobilized Hep-mod into PCL compared to NCC–Hep-mod1, resulted in a significant decrease in Young’s modulus (below the value characteristic of the pure PCL film), while the opposite trend was observed for composites obtained using NCC–APTES–PGlu2 as fillers. This may be attributed to the increased hydrophobicity of NCC–APTES–PGlu due to both the introduction of APTES and the higher content of hydrophobic moieties in PGlu compared to heparin (21 mol% benzyl groups vs. 3 mol% hexyl groups, respectively). The higher hydrophobicity of PGlu compared to Hep-mod also results in a larger hydrodynamic diameter of NCC–APTES–PGlu in an aqueous medium (Figure 4), which can be attributed not only to polyanion attachment, but also to more pronounced aggregation compared to other modified particles. As a result, hydrophobic NCC–APTES–PGlu particles are better distributed in the hydrophobic PCL matrix.

A rather significant decrease in all mechanical parameters was also observed for PCL/NCC–PGlu–Lys composites. The increase in particle size observed in the case of NCC–Hep and NCC–PGlu–Lys is only due to polyanion immobilization, which enhances the hydrophilicity of the filler, resulting in more prominent aggregation in the PCL matrix compared to NCC–APTES–PGlu.

In summary, it should be noted that incorporating of all types of NCC particles used in this work into PCL, even when Young’s modulus was increased, was accompanied by a noticeable decrease in other mechanical characteristics such as plasticity limit, strength and ultimate deformation compared to the characteristics of pure PCL film. Such influence of nanosized fillers of various types on the mechanical properties of polymeric materials has been reported in many publications devoted to the properties of polymer-inorganic nanocomposites [58,59,60]. This is explained by the processes of heterogenization of the composite material due to the introduction of nanoparticles into the polymer matrix, which, in turn, leads to the formation of phase boundaries in the polymer volume and, as a consequence, the appearance of internal stresses in the composite. The intensity of these processes depends on the degree of aggregation of the nanosized filler in the matrix volume. In the systems investigated in our work, this aggregation was quite pronounced due to the high hydrophilicity of the fillers (NCC and derivatives) and the high hydrophobicity of matrix polymer (PCL) (Figure 6 and Figure 7). Moreover, the aggregation was combined with pore-forming effects (Figure 8). As a result, a considerable decrease in the plasticity limit and fracture characteristics of the composite materials compared to the matrix polymer film was registered.

In general, the results obtained are in agreement with previously published findings on the preparation of PCL-based composites with NCC as fillers. For example, Habibi et al. showed that the modification of NCC with a hydrophobic polymer, namely PCL, significantly improved the Young’s modulus (tensile test) of PCL/NCC–PCL composite films compared to unfilled PCL and PCL/NCC composites [36]. However, other parameters were significantly decreased compared to unfilled PCL, and this trend was maintained when the filler content was increased from 10 to 40 wt%. A similar result was observed in the case of grafting PLA to NCC [35]. In that case, the PCL/NCC–PLA composite films showed an improvement in the Young’s modulus and a decrease in tensile strength and elongation at break compared to unfilled PCL. However, compared to the composite containing neat NCC, all tensile characteristics were improved.

Thus, in the case of modifying NCC with polyanions, the improvement of the Young’s modulus was observed only in those systems where NCC modification resulted in significant hydrophobization of the filler, promoting its homogeneous distribution in the matrix of hydrophobic PCL. Otherwise, the mechanical properties of the films remained close to those of the PCL composite filled with neat NCC. Despite the slight decrease in the values of tensile strength and elongation at break, in general, the mechanical parameters had values comparable to those determined for trabecular bone tissue in tensile tests. For example, for trabecular bone tissue, the values of elastic modulus in tensile tests range from a few tens to 800 MPa, depending on the area of bone sampling (tibia, femur, humerus, vertebrae, etc.) [61]. The elastic modulus values of 400–550 MPa obtained by us correspond to the values of the elastic modulus of the trabecular bone of the tibia, humerus, vertebrae, and patella. The tensile strength values of trabecular bone tissue lie in the range of 1–30 MPa [61]. In our case, the tensile strength values of 5–13 MPa obtained for composites correspond to the values inherent in the tibia, calcaneus, and vertebral bones. Meanwhile, it is known that the ultimate strain to failure in tensile mode for human femoral cortical bones does not exceed 2–3% [62]. From this point of view, the developed composites with values in the range of 10–15% are also acceptable. Hence, the developed composites meet the tensile properties of some bones.

### 3.3. Biological Evaluation of Composites

#### 3.3.1. *In Vitro* Biocompatibility

**Figure 10:**
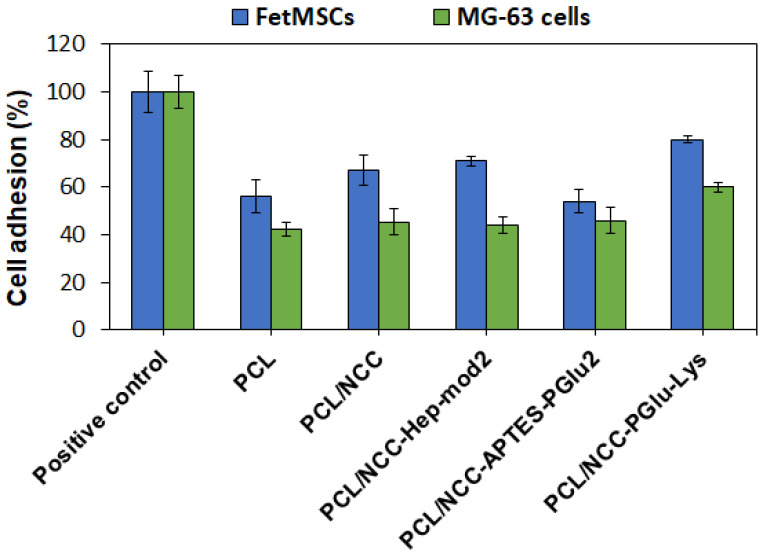
Adhesion of FetMSCs and MG-63 cells on surfaces of PCL and PCL-based composite films containing 10 wt% of neat and modified NCC as fillers (MTT, 72 h). Plastic surfaces of wells of adhesive 96-well cell culture plates were used as positive controls for cell adhesion.

All the components of the investigated composites, namely PCL [63], NCC [64], Hep [65] and PGlu [66], are known to be biocompatible. In this study, the effect of filler on the adhesion of FetMSCs and MG-63 cells on the surface of pure PCL and its composites with NCC was evaluated. In this case, 6 mm diameter film specimens were fixed onto the surface of wells of a non-adhesive 96-well plate. The plastic surface of the wells of the adhesive 96-well cell culture plate was used as a positive control for cell adhesion. The number of cells that adhered and survived after 72 h on the control surface was set to 100%. As shown in Figure 10, all PCL-based films showed lower cell adhesion compared to the positive control. Cell adhesion was found to be cell phenotype-dependent. In particular, the adhesion of FetMSCs was more pronounced than that of MG-63 cells. For the latter, cell adhesion was about 42–46% for all samples except for PCL/NCC–PGlu–Lys, for which it was 60%. In the case of FetMSCs, the lowest cell adhesion was observed for PCL and PCL/NCC–APTES–PGlu2 samples (54–56%) due to the hydrophobic nature of these materials. The presence of hydrophilic neat NCC and NCC–Hep-mod2 in the PCL matrix increased the adhesion capacity to 66–70%. However, the highest adhesion capacity (80%) was achieved with the PCL/NCC–PGlu–Lys composite, likely due to the presence of lysine residues in PGlu, which contains amino groups in the side chain that promote cell adhesion. Comparing the adhesion of the two cell lines used, the improved adhesion of MSCs holds greater practical importance in terms of the application of the developed materials as scaffolds. The adhesion of MSCs on the surface of scaffolds prior to their implantation promotes osteogenesis and, as a result, leads to faster regeneration of the bone tissue [27,67,68].

#### 3.3.2. MSCs Osteogenic Differentiation 

**Figure 11:**
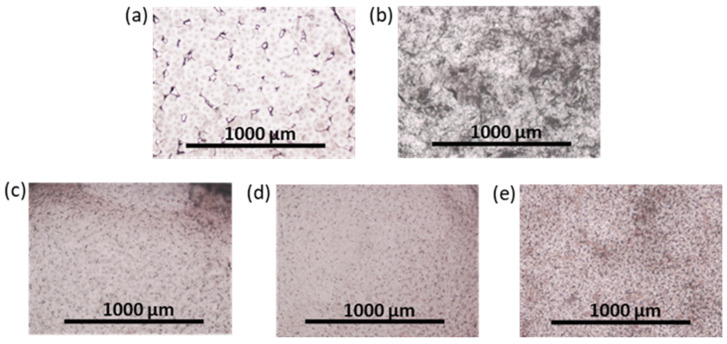
Images of surface of films after ALP staining (optical microscopy, ×4; two weeks of the osteogenic differentiation of FetMSCs on the films): (**a**) PCL, (**b**) PCL/NCC, (**c**) PCL/NCC–Hep-mod2, (**d**) PCL/NCC–APTES–PGlu2, and (**e**) PCL/NCC–PGlu–Lys. Amount of ALP is proportional to purple color intensity. Filler content in composites was 10 wt%.

FetMSCs adhered onto the surface of PCL and PCL-based composites with neat and modified NCC were subjected to osteogenic differentiation. After two weeks of incubation in an osteodifferentiation medium, qualitative detection of ALP expression, which is an early marker of osteogenesis [69], was performed. ALP is actively produced in osteoblasts and its appearance serves as a confirmation of MSC differentiation [70]. In this study, ALP detection was performed using the BCIP-NBT staining technique, which involves hydrolysis of a substrate (BCIP) by the ALP enzyme and subsequent oxidation of the intermediate blue product by NBT, leading to the formation of an insoluble purple product. The results obtained demonstrate that the most intense staining was observed for PCL/NCC–Hep-mod2 and PCL/NCC–PGlu–Lys composites, which also indicates their greater osteoconductive properties (Figure 11). Moreover, this result is consistent with the MTT data, which showed that more MSCs adhered onto the surface of these composites. At the same time, more uniform surface ALP staining was detected for PCL/NCC–PGlu–Lys composites.

**Figure 12:**
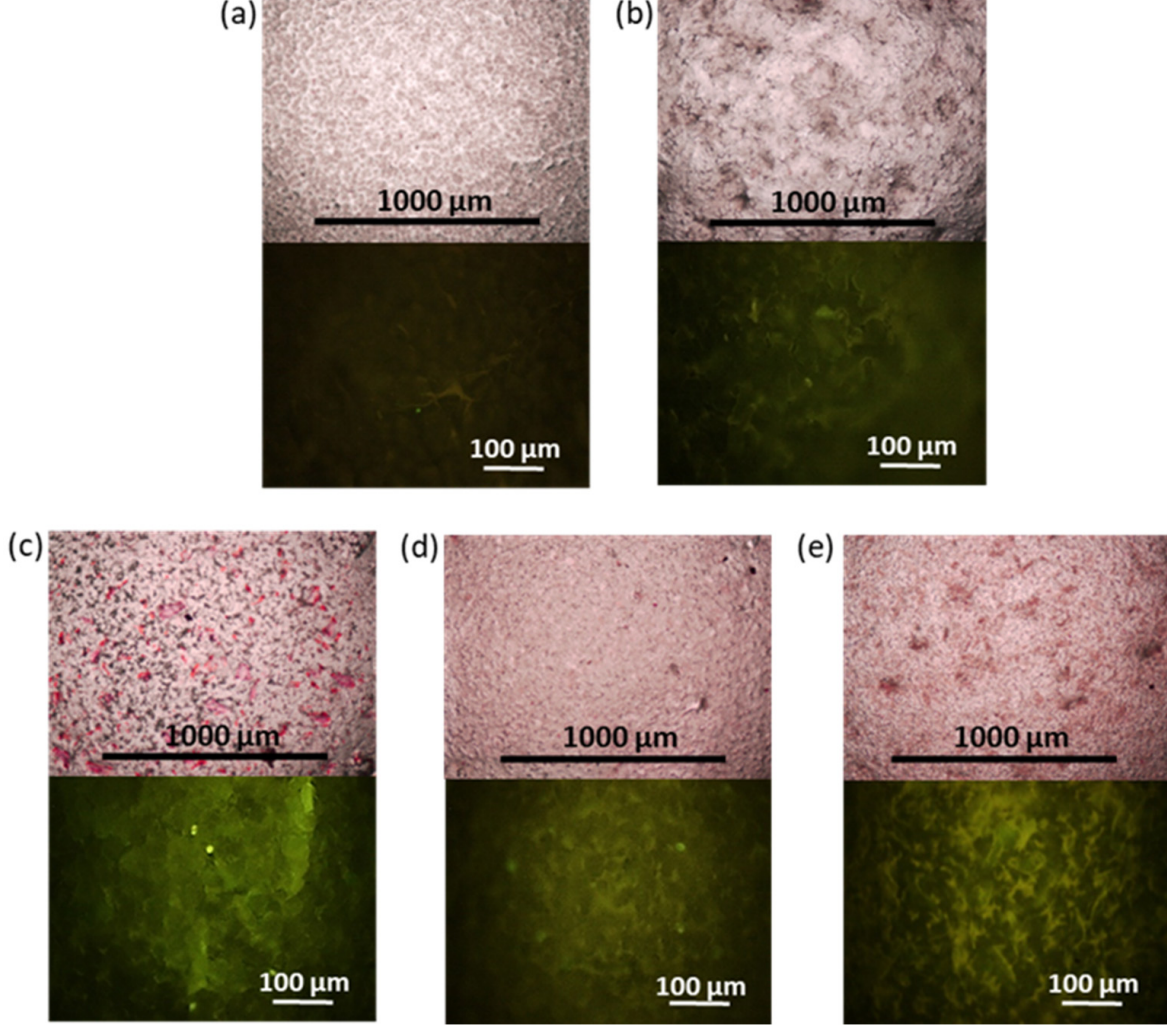
Images of surface of films after alizarin red S (top, optical microscopy, ×4) and calcein staining (fluorescein microscopy, ×20): (**a**) PCL/NCC, (**b**) PCL/NCC, (**c**) PCL/NCC–Hep-mod2, (**d**) PCL/NCC–APTES–PGlu2, and (**e**) PCL/NCC–PGlu–Lys. Filler content in composites was 10 wt%. Intensity of surface red or green staining is proportional to calcium deposits formed during osteogenic differentiation of FetMSCs (28 days).

The results of the ALP analysis after two weeks of osteogenic differentiation agree with the results of calcium deposit detection after four weeks of osteogenic differentiation of FetMSCs. Figure 12 shows images obtained by optical and fluorescence microscopy of the films stained with calcium-specific dyes, namely, alizarin red S and the fluorescent dye, calcein. As in the case of ALP detection, staining with alizarin red S and calcein revealed the most intense surface coloration, proportional to the formation of calcium deposits for PCL/NCC–Hep-mod2 and PCL/NCC–PGlu–Lys composites (Figure 12c,e). Thus, these composites can be considered the most promising in terms of further development of 3D scaffolds for bone regeneration. 

## 4. Conclusions

In this study, NCC was successfully functionalized with amphiphilic polyanions, such as modified heparin and poly(*αL*-glutamic acid). The modification of NCC was confirmed by a number of physicochemical methods, such as FTIR and solid-state ^13^C NMR spectroscopy, dynamic and electrophoretic light scattering as well as thermogravimetric analysis. The obtained specimens of modified NCC were used as fillers for the manufacturing of PCL-based films by solution casting. The use of a chloroform/acetonitrile mixture for the preparation of the films provided a homogeneous distribution of the modified NCC in the PCL matrix. The best mechanical properties were observed in the PCL films filled with NCC modified with hydrophobized PGlu bound after NCC silanization and functionalized heparin. The developed composites meet the tensile properties of some bones. *In vitro* biological evaluation showed low cell adhesion for PCL film and the PCL/NCC–APTES–PGlu composite, and high cell adhesion for composites containing NCC, NCC–Hep-mod or NCC–PGlu–Lys as fillers. However, only the last two fillers showed the best osteoconductive properties in PCL-based composites. The developed PCL and NCC-based composites functionalized with modified polyanions appear very promising for further investigation as 3D-printed scaffolds for bone tissue regeneration. 

## Figures and Tables

**Table 1:** Polyanions used for modification of NCC.

Abbreviation	Polymer Composition	Characteristics
Hep-mod	Hep-(EDA, Hex)	*M_n_* * = 14,700; *Đ* * = 1.6; *χ(Hex)* ** = 3 mol%; *χ(EDA)* = 8 mol%
PGlu	P[Glu-*co*-Glu(OBzl)]	*M_n_* ** = 8400; *Đ* * = 1.5; *χ(Bzl)* ** = 21 mol%
PGlu-Lys	P[Glu-*co*-Glu(Lys)-*co*-Glu(OBzl)]	*M_n_* ** = 8800; *Đ* * = 1.5; *χ(Bzl)* ** = 21 mol%; *χ(Lys)* ** = 5 mol%

* According to SEC analysis; ** According to ^1^H NMR spectroscopy. ^1^H NMR spectra and information on composition calculation can be found in Supplementary Materials.

## Data Availability

The data are available within the article and its Supplementary Materials.

## Supplementary Materials

The following supporting information can be downloaded at https://www.mdpi.com/article/10.3390/polym16131936/s1, Figure S1: ^1^H NMR spectra of (a) Hep-(EDA(Boc), Hex), (b) Hep-(EDA, Hex), (c) P[Glu-*co*-Glu(Lys)-*co*-Glu(OBzl)]; Figure S2: ^13^C NMR spectra of (a) NCC and NCC–Hep-mod2, (b) starting polymers and NCC–APTES–PGlu2, (c) starting polymers and NCC–PGlu–Lys; Table S1: Values of *τ*_5_ and *τ*_10_ for neat and modified NCC; Figure S3: Tensile stress-strain curves of film composite materials based on PCL and unmodified and modified NCC. References [71,72,73] are cited in the Supplementary Materials.
