# Supplementary material for: Chemical Modification of Nanocrystalline Cellulose for Manufacturing of Osteoconductive Composite Materials
Source: Polymers (Basel). 2024 Jul 6;16(13):1936. doi: 10.3390/polym16131936 (PMC11244019; doi:10.3390/polym16131936)
Supplement: Supplementary file 1 [file polymers-16-01936-s001.zip › polymers-3037473-supplementary.pdf]

Supplementary Materials

Chemical Modification of Nanocrystalline Cellulose for Manufacturing of Osteoconductive Composite Materials

Olga Solomakha [1], Mariia Stepanova [1,*], Anatoliy Dobrodumov [1], Iosif Gofman [1], Yulia Nashchekina [2], Alexey Nashchekin [3] and Evgenia Korzhikova-Vlakh [1,*]

1 Institute of Macromolecular Compounds, Russian Academy of Sciences, St. Petersburg 199004, Russia
2 Institute of Cytology, Russian Academy of Sciences, St. Petersburg 194064, Russia
3 Ioffe Institute, St. Petersburg 194021, Russia
* Correspondence: maristepanova@hq.macro.ru (M.S.); vlakh@hq.macro.ru (E.K.-V.)

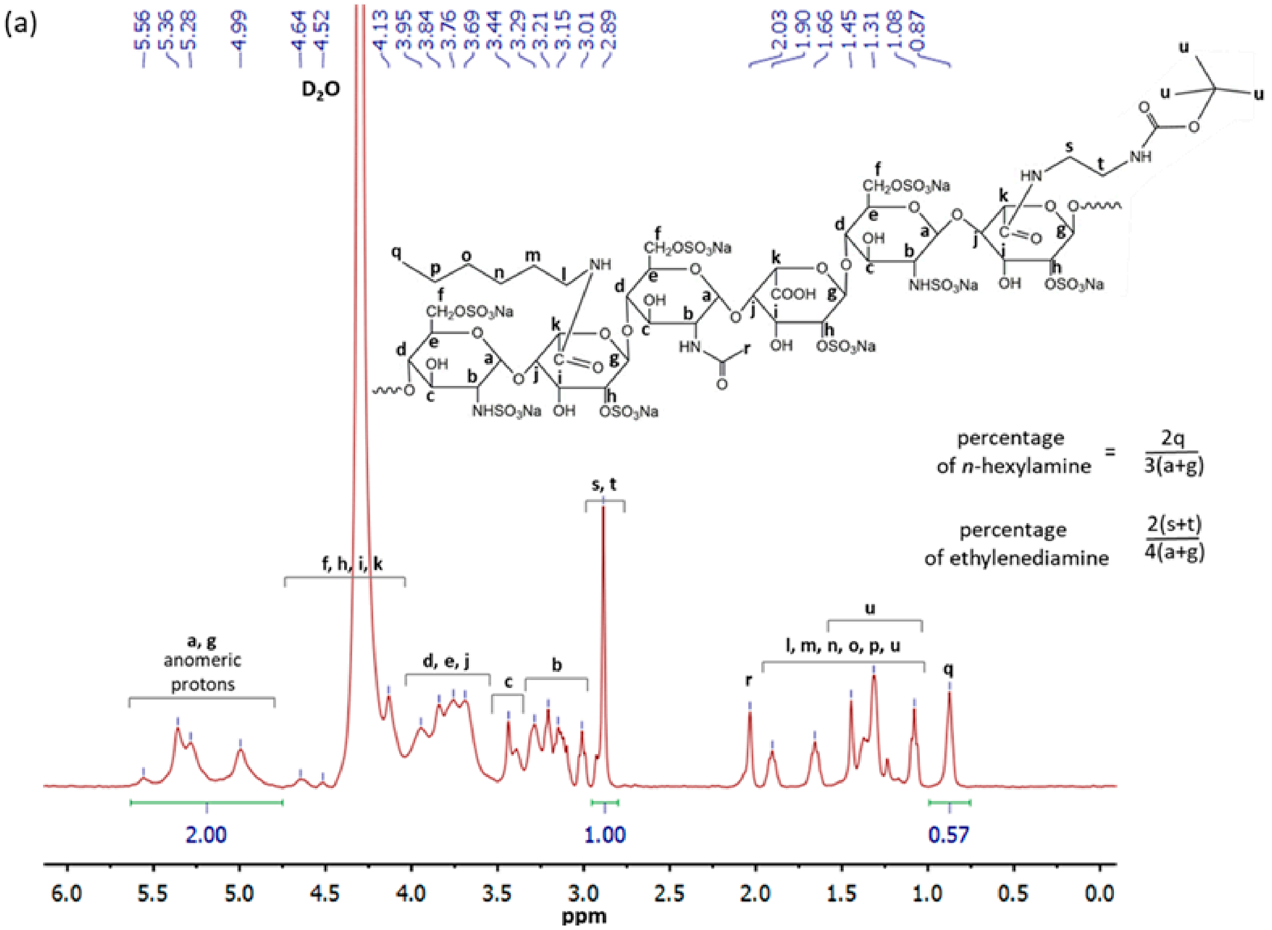

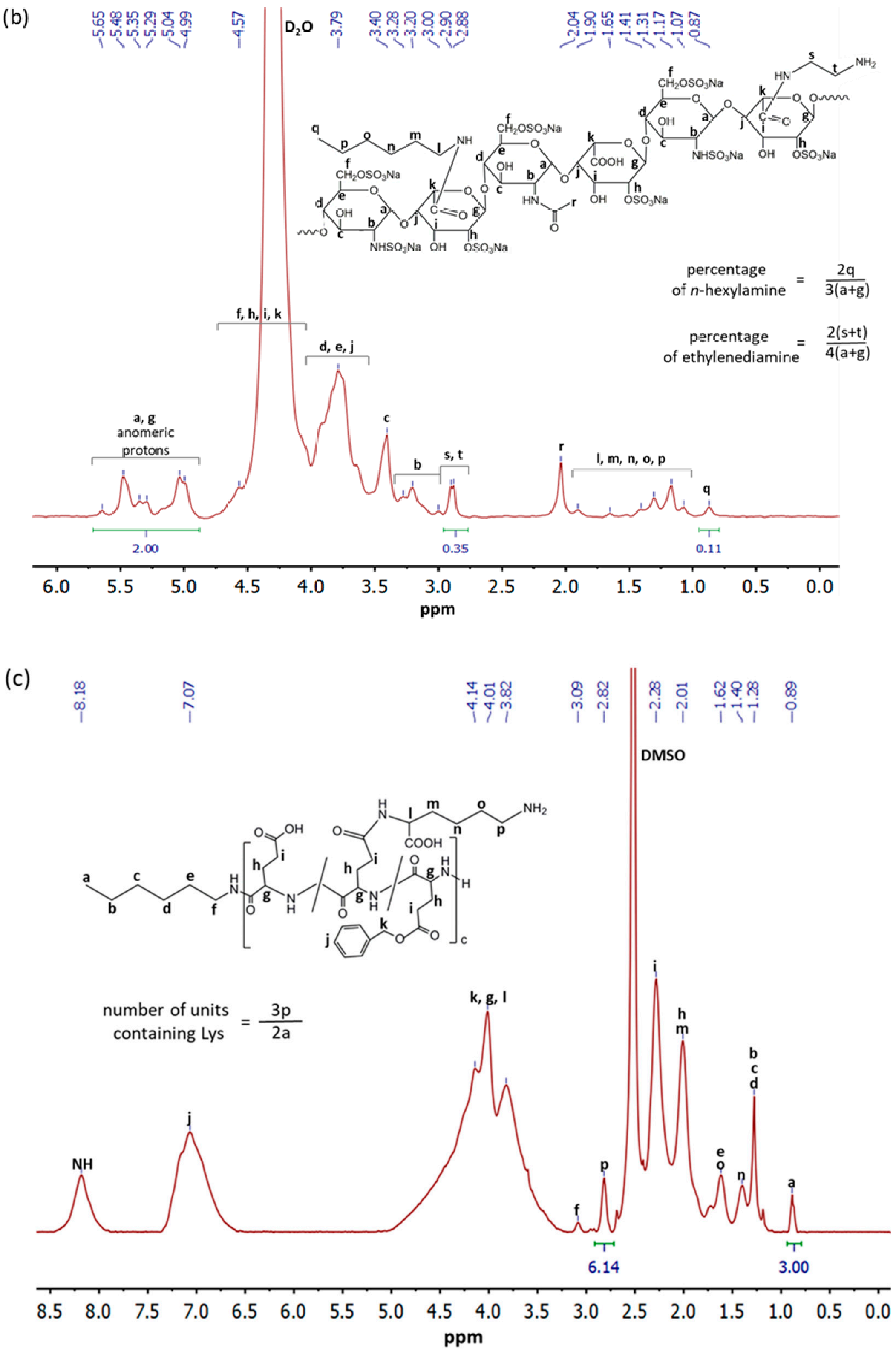

Figure S1. ^{1}H NMR spectra of (**a**) Hep-(EDA(Boc), Hex), (**b**) Hep-(EDA, Hex), (**c**) P[Glu-*co*-Glu(Lys)-*co*-Glu(OBzl)]. Identification of signals for heparin, poly(glutamic acid), *n*-hexilamine, *N*-(tert-butoxycarbonyl)-ethylenediamine and ethylenediamine was carried out according to literature data [40,56,57,71,72]. The contnet of L-lysine in NCC-PGlu-Lys is calculated using the equation χ(Lys) = (number of units containing Lys)/(degree of polymerization of PGlu) and is 5 mol%.

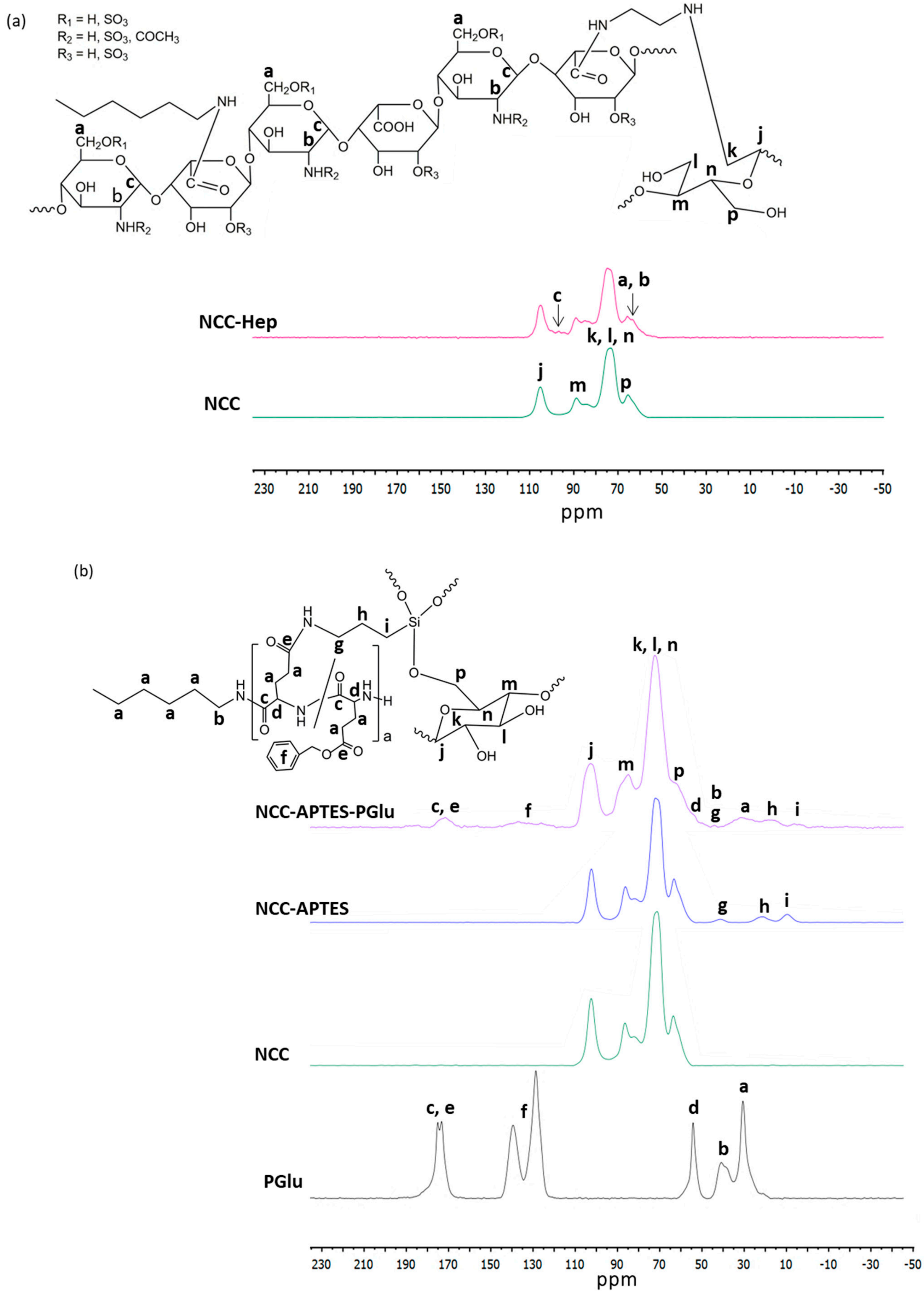
(a)
R1 = H, SO3
R2 = H, SO3, COCH3
R3 = H, SO3
NCC-Hep
NCC
ppm
(b)
NCC-APTES-PGlu
NCC-APTES
NCC
PGlu
ppm

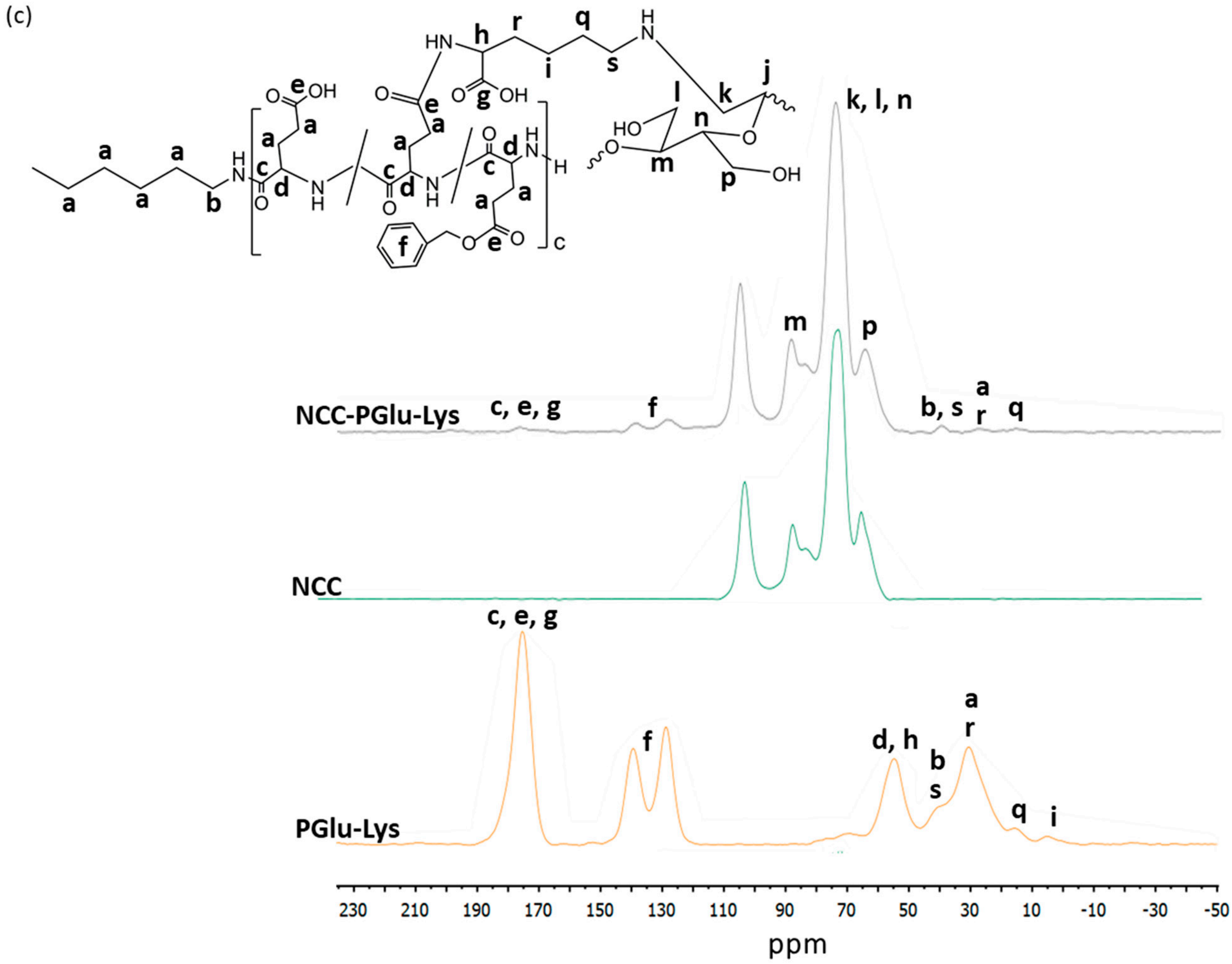

Figure S2. ^{13}C NMR spectra of (**a**) NCC and NCC-Hep-mod2, (**b**) starting polymers and NCC-APTES-PGlu2, (**c**) starting polymers and NCC-PGlu-Lys. Identification of signals for heparin, poly(glutamic acid), nanocrystalline cellulose was carried out according to literature data [40,49,57,73].

Table S1. Values of τ_5 and τ_{10} for the neat and modified NCC.

Specimen	τ_5 (°C)*	τ_{10} (°C)*
NCC	212	237
NCC-Hep1	249	271
NCC-APTES-PGlu1	218	257
NCC-PGlu-Lys	216	239

* The relative standard deviation was 1-2%.

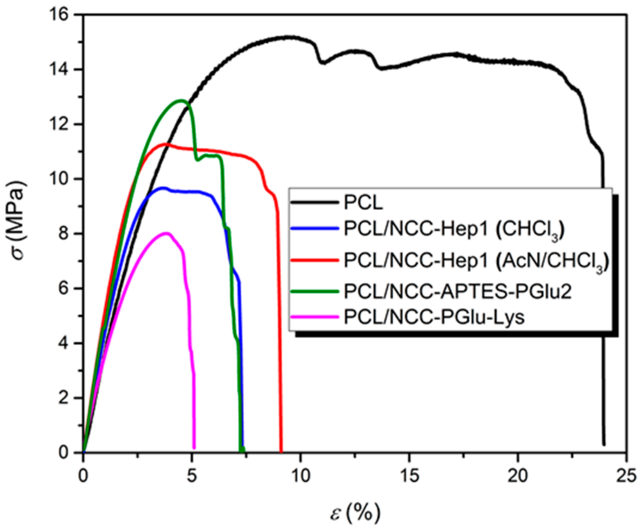

Figure S3. Tensile stress-strain curves of film composite materials based on PCL and unmodified and modified NCC. Filler content 10 wt%.
